# Supplementary material for: METASPACE-ML: Context-specific metabolite annotation for imaging mass spectrometry using machine learning
Source: Nat Commun. 2024 Oct 22;15:9110. doi: 10.1038/s41467-024-52213-9 (PMC11496635; doi:10.1038/s41467-024-52213-9)
Supplement: Supplementary file 3 — Description of Additional Supplementary Files [file 41467_2024_52213_MOESM3_ESM.pdf]

Description of Additional Supplementary Files:

Supplementary Data 1: Curated organism metadata. Mapping between organism metadata field and their corresponding species/genus and kingdom

Supplementary Data 2: Classification of all public datasets. List of all public datasets classified by their curated metadata (source data for Figures 2A,B)

Supplementary Data 3: Classification of datasets by acquisition geometry. Classification of datasets into either having regular or irregular ion images.

Supplementary Data 4: Exclusion criteria for public dataset. Binary classification of all public datasets for the 3 criteria used to exclude low-quality datasets (see Exclusion of low-quality datasets, Methods)

Supplementary Data 5: METASPACE datasets IDs and metadata for training and testing datasets. The public datasets can be accessed and downloaded by their ID either through METASPACE webapp by opening the URL https://metaspace2020.eu/dataset/ e.g. https://metaspace2020.eu/dataset/2018-04-24_18h54m28s for the dataset ID 2018-04-24_18h54m28s or through the METASPACE API (https://metaspace2020.readthedocs.io).

Supplementary Data 6: METASPACE datasets IDs and metadata for database comparison. The public datasets can be accessed and downloaded by their ID either through METASPACE webapp by opening the URL https://metaspace2020.eu/dataset/ e.g. https://metaspace2020.eu/dataset/2018-04-24_18h54m28s for the dataset ID 2018-04-24_18h54m28s or through the METASPACE API (https://metaspace2020.readthedocs.io).

Supplementary Data 7: Metabolite classification enrichment results for test datasets. Overrepresentation analysis results for each test dataset against HMDB subclasses as background (source data for Figures 7C,D).

Supplementary Data 8: METASPACE datasets IDs and metadata for LC-MS/MS bulk validation. The public datasets can be accessed and downloaded by their ID either through METASPACE webapp by opening the URL https://metaspace2020.eu/dataset/ e.g. https://metaspace2020.eu/dataset/2018-04-24_18h54m28s for the dataset ID 2018-04-24_18h54m28s or through the METASPACE API (https://metaspace2020.readthedocs.io).

Supplementary Data 9: LC-MS/MS bulk validation results. LC-MS/MS bulk validation data

Supplementary Data 10: Target-decoy ranking and FDR example. Metrics, raw scores and corresponding FDR scores for a specific group (+Na adducts) within a particular dataset (https://metaspace2020.eu/dataset/2018-12-14_16h34m31s) .
