# Supplementary material for: METASPACE-ML: Context-specific metabolite annotation for imaging mass spectrometry using machine learning
Source: Nat Commun. 2024 Oct 22;15:9110. doi: 10.1038/s41467-024-52213-9 (PMC11496635; doi:10.1038/s41467-024-52213-9)
Supplement: Supplementary file 5 — Reporting Summary [file 41467_2024_52213_MOESM5_ESM.pdf]

nature portfolio

Corresponding author(s): Theodore Alexandrov

Last updated by author(s): Aug 19, 2024



# Reporting Summary

Nature Portfolio wishes to improve the reproducibility of the work that we publish. This form provides structure for consistency and transparency in reporting. For further information on Nature Portfolio policies, see our Editorial Policies and the Editorial Policy Checklist.

## Statistics

For all statistical analyses, confirm that the following items are present in the figure legend, table legend, main text, or Methods section.

| n/a | Confirmed | |
|---|---|---|
| [ ] | [x] | The exact sample size ($n$) for each experimental group/condition, given as a discrete number and unit of measurement |
| [ ] | [x] | A statement on whether measurements were taken from distinct samples or whether the same sample was measured repeatedly |
| [ ] | [x] | The statistical test(s) used AND whether they are one- or two-sided<br>*Only common tests should be described solely by name; describe more complex techniques in the Methods section.* |
| [x] | [ ] | A description of all covariates tested |
| [ ] | [x] | A description of any assumptions or corrections, such as tests of normality and adjustment for multiple comparisons |
| [ ] | [x] | A full description of the statistical parameters including central tendency (e.g. means) or other basic estimates (e.g. regression coefficient) AND variation (e.g. standard deviation) or associated estimates of uncertainty (e.g. confidence intervals) |
| [ ] | [x] | For null hypothesis testing, the test statistic (e.g. $F$, $t$, $r$) with confidence intervals, effect sizes, degrees of freedom and $P$ value noted<br>*Give P values as exact values whenever suitable.* |
| [x] | [ ] | For Bayesian analysis, information on the choice of priors and Markov chain Monte Carlo settings |
| [x] | [ ] | For hierarchical and complex designs, identification of the appropriate level for tests and full reporting of outcomes |
| [x] | [ ] | Estimates of effect sizes (e.g. Cohen's $d$, Pearson's $r$), indicating how they were calculated |

*Our web collection on statistics for biologists contains articles on many of the points above.*

## Software and code

Policy information about availability of computer code

| | |
|---|---|
| Data collection | METASPACE-API (https://metaspace2020.readthedocs.io/en/latest/) was used to reprocess and retrieve the public data from METASPACE. |
| Data analysis | Software packages and modules used in this study include: R (v4.1.2), tidyverse (v1.3.1), ComplexHeatmap (v2.10.0), ggpubr (v0.4.0), ggsankey (v0.0.99999), ggrepel (v0.9.1), ggridges (v0.5.3), ggstatsplot (v0.9.5), RColorBrewer (v1.1.2), DataExplorer (v0.8.2), cowplot (v1.1.1), cutpointr (v1.1.2), GGally (v2.1.2), rstatix (v0.7.0), ggbiplot (v0.55), M3C (v1.16.0), pROC (v1.18.0), hexbin (v1.28.2), Python (3.8.13), pandas (v1.2.0), numpy (v1.19.5), sklearn (v0.23.2), catboost (v1.0.3), shap (0.40.0), metaspace (v2.0.0). |

For manuscripts utilizing custom algorithms or software that are central to the research but not yet described in published literature, software must be made available to editors and reviewers. We strongly encourage code deposition in a community repository (e.g. GitHub). See the Nature Portfolio guidelines for submitting code & software for further information.

## Data

Policy information about availability of data

All manuscripts must include a data availability statement. This statement should provide the following information, where applicable:

- Accession codes, unique identifiers, or web links for publicly available datasets
- A description of any restrictions on data availability
- For clinical datasets or third party data, please ensure that the statement adheres to our policy

All datasets analysed in this study are publicly available. The public training and testing datasets are available on METASPACE [https://metaspace2020.eu/] and more information on each dataset can be accessed from Supplementary Table 5. Context-specific datasets can be accessed from the Shiny-based web app (https://

t.ly/q-nb5). The LC-MS/MS data used for validation are publicly available at the MetaboLights repository under accession code MTBLS378 [http://www.ebi.ac.uk/metabolights/MTBLS378]. Raw source data is also available at BioStudies repository under accession code S-BIAD1283 [https://www.ebi.ac.uk/biostudies/bioimages/studies/S-BIAD1283]

## Human research participants

Policy information about studies involving human research participants and Sex and Gender in Research.

| | |
|---|---|
| Reporting on sex and gender | There was no human research participants involved in this study |
| Population characteristics | There was no human research participants involved in this study |
| Recruitment | There was no human research participants involved in this study |
| Ethics oversight | There was no human research participants involved in this study |

Note that full information on the approval of the study protocol must also be provided in the manuscript.

# Field-specific reporting

Please select the one below that is the best fit for your research. If you are not sure, read the appropriate sections before making your selection.

[x] Life sciences [ ] Behavioural & social sciences [ ] Ecological, evolutionary & environmental sciences

For a reference copy of the document with all sections, see nature.com/documents/nr-reporting-summary-flat.pdf

# Life sciences study design

All studies must disclose on these points even when the disclosure is negative.

| | |
|---|---|
| Sample size | We experimented with various sample sizes (ranging from 10-50 datasets per context) for model training, and we chose a context size of 30 because of minimal difference between training and validation error. For testing datasets, 30 datasets was chosen in order to to strike a balance where including more datasets could lead to less diversity of contexts, whereas including fewer datasets might risk compromising statistical reliability due to a smaller sample size potentially leading to less robust evaluation. |
| Data exclusions | We have developed the following criteria to exclude low-quality datasets from training and testing sets: (1) The number of annotations at FDR 20% is less than 10 for each possible target adduct and annotation database combination, which resulted in the exclusion of 1,418 datasets; (2) The median (across pixels) number of m/z peaks with non-zero intensity > 50,000, this led to exclusion of 6 datasets; (3) The proportion_overlap score > 0.5 (see Methods) which led to the exclusion of 127 datasets. |
| Replication | Replication is not relevant to this study since the datasets are publicly available, and replicates are not required for model training. |
| Randomization | Randomization is not relevant to this study. |
| Blinding | Blinding is not relevant to this study. |

# Reporting for specific materials, systems and methods

We require information from authors about some types of materials, experimental systems and methods used in many studies. Here, indicate whether each material, system or method listed is relevant to your study. If you are not sure if a list item applies to your research, read the appropriate section before selecting a response.

## Materials & experimental systems

| n/a | Involved in the study | |
|---|---|---|
| [x] | [ ] | Antibodies |
| [x] | [ ] | Eukaryotic cell lines |
| [x] | [ ] | Palaeontology and archaeology |
| [x] | [ ] | Animals and other organisms |
| [x] | [ ] | Clinical data |
| [x] | [ ] | Dual use research of concern |

## Methods

| n/a | Involved in the study | |
|---|---|---|
| [x] | [ ] | ChIP-seq |
| [x] | [ ] | Flow cytometry |
| [x] | [ ] | MRI-based neuroimaging |
